# Comparative assessment of genetic diversity in *Sesamum indicum* L. using RAPD and SSR markers

**DOI:** 10.1007/s13205-016-0578-4

**Published:** 2017-04-08

**Authors:** Aejaz Ahmad Dar, Sushma Mudigunda, Pramod Kumar Mittal, Neelakantan Arumugam

**Affiliations:** Department of Biotechnology, School of Life Sciences, Pondicherry University, Puducherry, 605014 India

**Keywords:** *Sesamum indicum*, RAPD, SSR, Genetic diversity, Polymorphic information content, Seed coat colour

## Abstract

Sesame (*Sesamum indicum* L.) is an ancient oilseed crop known for its nutty seeds and high-quality edible oil. It is an unexplored crop with a great economic potential. The present study deals with assessment of genetic diversity in the crop. Twenty two RAPD and 18 SSR primers were used for analysis of the 47 different sesame accessions grown in different agroclimatic zones of India. A total of 256 bands were obtained with RAPD primers, of which 191 were polymorphic. SSR primers gave 64 DNA bands, of which all of were polymorphic. The Jaccardʼs similarity coefficient of RAPD, SSR, and pooled RAPD and SSR data ranged from 0.510 to 0.885, 0.167 to 0.867, and 0.505 to 0.853, respectively. Maximum polymorphic information content was reported with SSRs (0.194) compared to RAPDs (0.186). Higher marker index was observed with RAPDs (1.426) than with SSRs (0.621). Similarly, maximum resolving power was found with RAPD (4.012) primers than with SSRs (0.884). The RAPD primer RPI-B11 and SSR primer S16 were the most informative in terms of describing genetic variability among the varieties under study. At a molecular level, the seed coat colour was distinguishable by the presence and absence of a group of marker amplicon/s. White and brown seeded varieties clustered close to each other, while black seeded varieties remained distanced from the cluster. In the present study, we found higher variability in *Sesamum indicum* L. using RAPD and SSR markers and these could assist in DNA finger printing, conservation of germplasm, and crop improvement.

**Electronic supplementary material:**

The online version of this article (doi:10.1007/s13205-016-0578-4) contains supplementary material, which is available to authorized users.

## Introduction

*Sesamum indicum* L. (Pedaliaceae), commonly known as sesame or Gingelly, is an ancient oilseed crop cultivated in almost all continents. It is a source of high-quality edible oil that contains, in addition, medicinally important antioxidant lignans, namely, sesamin, sesamolin, and sesamol (Anilakumar et al. 2010; Dar et al. 2014). Lignans impart resistance to rancidity of the oil and enhance tumor inhibiting properties of certain drugs (Sacco et al. 2007; Dar and Arumugam 2013; Dar et al. 2015). India, Sudan, and China are the major sesame producing countries. However, the global per unit area production of sesame seed, especially in developing countries, is far less (FAO STAT 2014). The low yield has been attributed to indeterminate growth, uneven ripening of capsules, absence of non-shattering types, and biotic and abiotic stresses (Ashri 1998). Importance of crop genetic diversity in the era of post green revolution and threat by change cannot be over emphasized (Abberton et al. 2015). A focused research on genetic diversity and breeding of sesame is needed to improve its worldwide productivity.

Morpho-agronomic traits have been the prime variables used for studying genetic diversity (Liu 1997; Bisht et al. 1998). Selection of plant varieties based on these traits alone has proved to be ineffective due to problems of low heritability, strong influence of the environment, and genetic complexity. Fortunately, advances in molecular technologies have enabled proper identification, selection, and use of germplasm in crop improvement programmes (Spandana et al. 2012). DNA markers in particular are useful and reliable as they remain stable under different environmental conditions (Ferdinandez et al. 2001). Random amplified polymorphic DNA (RAPD) is the simplest of all molecular techniques enabling quick detection of polymorphisms at a number of loci with only small amounts (nanogram) of genomic DNA required (Bhat et al. 1999; Salazar et al. 2006; Pham et al. 2011). RAPD technique first reported by Welsh and McClelland (1990) and Williams et al. (1990) has been routinely used for genetic analysis in a vast array of crops, including foxtail millet, sunflower, birch, soybean, and oilseed rape (Schontz and Rether 1999; Popov et al. 2002; Xu and Gai 2003; Zeng et al. 2003; Yu et al. 2005). Simple sequence repeat (SSR) is yet another popular DNA marker used in the assessment of the genetic diversity, phylogenetic relationships, and population structures in crops. SSR markers are tandem repeats of short nucleotide sequences of about one-to-six bases that have revealed a high polymorphism in several crops, including barley (Malysheva-Otto et al. 2006), perilla (Park et al. 2008), and rice (Cui et al. 2010). The polymorphism with this marker arise either due to polymerase slippage during DNA replication or unequal crossing over (Levinson and Gutman 1987). The SSR has advantage of identifying many alleles at a single locus, extensive genome coverage, co-dominance, and repeatability, can be carried out with just a small quantity of DNA (ng), and avoids radioactivity (Abdelkrim et al. 2009; Allentoft et al. 2009). The SSR markers have also proved useful in the preparation of genetic map by linking it with other markers (Nimmakayala et al. 2005).

As compared to many crop plants, information on molecular characterization of genetic diversity in sesame has been very meagre. The molecular techniques of RAPD (Bhat et al. 1999; Ercan et al. 2004; Kumar and Sharma 2009; Pham et al. 2011; Akbar et al. 2011; Mahdizadeh et al. 2012), AFLP (amplified fragment length polymorphism) (Laurentin and Karlovsky 2006, 2007), ISSR (inter-simple sequence repeats) (Kim et al. 2002; Parsaeian et al. 2011; Kumar and Sharma 2011; Nyongesa et al. 2013; Woldesenbet et al. 2015), SSR (Spandana et al. 2012; Dixit et al. 2005; Cho et al. 2011; Wang et al. 2012; Yepuri et al. 2013; Wei et al. 2014; Surapaneni et al. 2014; Uncu et al. 2015; Dossa et al. 2016; Sehr et al. 2016), and SRAP (sequence-related amplified polymorphism) (Li and Quiros 2001; Li et al. 2007; Zhang et al. 2010) have been used to evaluate the genetic diversity in sesame of different origins. Morphologically, Indian collection of sesame is characterized by variable forms being cultivated in different agroclimatic zones. The molecular data on the diversity of Indian germplasm are very limited. In this paper, we describe comparative evaluation of molecular genetic diversity of the sesame varieties of Indian origin using RAPD and SSR markers. The results are discussed in the light of developing strategy for breeding and conservation of the crop.

## Materials and methods

### Plant material

The 47 improved varieties of sesame germplasm used in the study are presented in Table 1. The seeds of the germplasm were procured from the National Bureau of Plant Genetic Resources (NBPGR), New Delhi, and the crop was raised and maintained by growing in the experimental garden of our department. The members of the germplasm differed in their seed colour and the region of their cultivation.

**Table 1:** List and details of improved varieties of *Sesamum indicum* L. used in the present study

Population	Variety name	Seed coat colour	Location of cultivation^a^
1	AMRIT	Brown	Andhra pradesh
	YLM17	Brown	Andhra pradesh
	CHANDANA	Brown	Andhra pradesh
	RAJESWARI	White	Andhra pradesh
2	KRISHNA	Black	Bihar
	TARUN	White	Bihar
3	GT10	Black	Gujarat
	GT1	White	Gujarat
	GT2	White	Gujarat
4	SVPRI	White	Karnataka
	E8	White	Karnataka
	DS1	White	Karnataka
5	TKG22	White	Madhya pradesh
	JTS8	White	Madhya pradesh
	JT7	White	Madhya pradesh
	TKG55	White	Madhya pradesh
	N32	White	Madhya pradesh
6	JLT1	Black	Maharashtra
	N8	Brown	Maharashtra
	JLT7	White	Maharashtra
	AKT64	White	Maharashtra
	JLT26	White	Maharashtra
	PHULETIL	White	Maharashtra
7	PRACHI	Black	Orissa
	UMA	Brown	Orissa
	NIRMALA	Brown	Orissa
	VINAYAK	Brown	Orissa
	KALIKA	Brown	Orissa
8	TC25	White	Punjab
	PBTIL	White	Punjab
	TC289	White	Punjab
9	RT46	White	Rajasthan
	RT127	White	Rajasthan
	RT103	White	Rajasthan
	RT125	White	Rajasthan
10	TMV3	Black	Tamil nadu
	VRI1	Brown	Tamil nadu
	TMV4	Brown	Tamil nadu
	TMV5	Brown	Tamil nadu
	TMV6	Brown	Tamil nadu
11	T78	White	Uttar pradesh
	SHEKHAR	White	Uttar pradesh
	T13	White	Uttar pradesh
	T12	White	Uttar pradesh
	PRAGATHI	White	Uttar pradesh
12	XLM19	Brown	NA
	FFAT-08-22	White	NA

*NA* not available

^a^Source: Hand book of Agriculture, ICAR, New Delhi

### DNA isolation and quantification

Fresh leaves from healthy plants were used for DNA isolation. Approximately 1 g of leaf tissue taken in a pestle and mortor and ground into a fine powder in liquid nitrogen and total genomic DNA was isolated following CTAB method (Murray and Thompson 1980), and the DNA isolated was dissolved in TE buffer (10 mM Tris, 1 mM EDTA, pH 8.0) and stored at −20 °C until use. DNA concentration was determined using Nanodrop (Thermo Scientific Nanodrop 2000 Spectrophotometer), and the purity was confirmed by electrophoresis on 0.8% nuclease-free and protease-free agarose gel run with a power pack set at 80 V for 1 h in 1× TAE buffer consisting of 0.04 M Tris base, 17.4 M Glacial acetic acid, 0.001 M EDTA.

### RAPD/SSR primers and PCR conditions

For RAPD analysis, 22 random primers of RPI-B series procured from GeNei™, Bangalore were used (Table 2). The primers were 10 bp in length with partial specificity to detect polymorphism in plants in detection of polymorphisms. Some preliminary experiments were carried out to arrive at the optimal condition for the RAPD-PCR. The results showed that a 25 µL reaction mix containing of 2.5 µL of 10× PCR buffer, 1 µL of 10 mM dNTP mix, 100 ng of RAPD primer, 0.5 µL Taq DNA polymerase (3U/µl), and 5 µL of template DNA (10 ng/µl) was optimal. PCR was done in an Eppendorf Mastercycler gradient (Germany) with an initial extended denaturation step at 94 °C for 5 min followed by 8 cycles of denaturation (94 °C for 45 s), primer annealing (35 °C for 1 min), and elongation (72 °C for 1.5 min), followed again by 35 cycles of 94 °C for 45 s, 38 °C for 1 min, and 72 °C for 1 min with final extended elongation step at 72 °C for 10 min.

**Table 2:** Marker attributes of RAPD primers used in the genetic assessment of *Sesamum indicum* L.

Primer	Sequence (5′–3′)	Fragment size (bp)	NB	NU	NP	FP	FA	GD	PIC	EMR	MI	Rp
RPI-B3	AAGCGACCTG	250–2500	13	0	12	0.923	0.789	0.299	0.243	11.076	2.691	5.489
RPI-B4	AATCGCGCTG	150–1750	13	0	12	0.923	0.759	0.328	0.262	11.076	2.902	6.255
RPI-B5	AATCGGGCTG	250–2000	15	1	12	0.800	0.810	0.248	0.200	9.600	1.920	5.702
RPI-B7	ACATCGCCCA	200–1250	9	0	5	0.556	0.863	0.197	0.159	2.780	0.442	2.468
RPI-B8	ACCACCCACC	800–1000	2	0	1	0.500	0.957	0.078	0.072	0.500	0.036	0.170
RPI-B9	ACCGCCTATG	180–1500	15	0	14	0.933	0.790	0.293	0.238	13.062	3.109	6.298
RPI-B10	ACGATGAGCG	250–1750	12	0	10	0.833	0.895	0.180	0.159	8.330	1.324	2.511
RPI-B11	ACGGAAGTGG	250–1600	11	0	11	1.000	0.710	0.368	0.292	11.000	3.212	6.383
RPI-B12	ACGGCAACCT	220–1300	12	0	9	0.750	0.782	0.275	0.216	6.750	1.458	5.234
RPI-B13	ACGGCAAGGA	225–3000	12	1	12	1.000	0.848	0.225	0.190	12.000	2.280	3.660
RPI-B14	ACTTCGCCAC	225–1300	9	0	8	0.889	0.775	0.310	0.249	7.112	1.771	4.043
RPI-B15	AGCCTGAGCC	350–1750	8	0	6	0.750	0.838	0.237	0.193	4.500	0.869	2.596
RPI-B16	AGGCGGCAAG	300–1300	8	0	7	0.875	0.747	0.335	0.267	6.125	1.635	4.043
RPI-B17	AGGCGGGAAC	290–1200	13	0	11	0.846	0.787	0.289	0.232	9.306	2.159	5.532
RPI-B18	AGGCTGTGTC	490–1500	9	2	6	0.667	0.894	0.151	0.124	4.002	0.496	1.915
RPI-B19	AGGTGACCGT	210–2000	13	2	9	0.692	0.866	0.179	0.145	6.228	0.903	3.489
RPI-B20	AGTCCGCCTC	120–1500	10	0	7	0.700	0.883	0.164	0.137	4.900	0.671	2.340
RPI-B21	CACGAACCTC	300–1200	11	1	5	0.455	0.938	0.098	0.082	2.275	0.187	1.362
RPI-B22	CATAGAGCGG	100–1600	18	1	12	0.667	0.826	0.214	0.170	8.004	1.361	6.255
RPI-B23	CCAGCAGCTA	100–1500	16	1	8	0.500	0.872	0.180	0.143	4.000	0.572	4.085
RPI-B24	CCAGCCGAAC	150–2000	13	0	5	0.385	0.895	0.143	0.115	1.925	0.221	2.723
RPI-B25	GAGCGCCTTC	250–2250	14	0	9	0.643	0.796	0.256	0.201	5.787	1.163	5.702
Total			256.00	9.00	191.00	–	–	–	–	–	–	–
Mean			11.636	0.409	8.682	0.740	0.833	0.229	0.186	6.834	1.426	4.012
Minimum			2.000	0.000	1.000	0.385	0.710	0.078	0.072	0.500	0.036	0.170
Maximum			18.000	2.000	14.000	1.000	0.957	0.368	0.292	13.062	3.212	6.383

*PIC* polymorphic information content, *EMR* effective multiplex ratio, *MI* marker index, *RP* resolving power

*NB* number of bands, *NU* number of unique bands, *NP* number of polymorphic bands, *FP* fraction of polymorphism, *FA* frequency of alleles, *GD* gene diversity

Eighteen pairs of SSR primers selected from previous reports (Dixit et al. 2005; Wei et al. 2011) and procured by synthesis from Sigma Aldrich (India). The sequences of the primers are given in Table 3. PCR was carried out in a reaction volume of 20 µl containing 1× PCR buffer (100 mM Tris–HCl, pH 8.3, 500 mM KCl, 15 mM MgCl_2,_ and 0.01% gelatin), 0.2 mM dNTP mix, 1 µM of each of forward and reverse primer, 1U Taq Polymerase, and 200 ng of template DNA. Reaction mixture was diluted to final volume by nuclease-free water. The amplification was carried out using the PCR programme: 94 °C for 3 min, then 30 cycles each of 94 °C for 30 s, 55 °C for 45 s, and 72 °C for 1 min followed by 10 cycles of 94 °C for 30 s, 53 °C for 45 s, and 72 °C for 1 min and a final extension of 72 °C for 10 min.

**Table 3:** Marker attributes of SSR primers used in the genetic assessment of *Sesamum indicum* L.

Primer code	Primer sequence	Allele size (bp)	Na	NUA	NPA	FP	FA	GD	PIC	EMR	MI	RP
S1	F: 5′-TCATATATAAAAGGAGCCCAAC–3′ R: 5′-GTCATCGCTTCTCTCTTCTTC–3′	100–700	5	2	5	1	0.936	0.117	0.108	5	0.541	0.638
S2	F: 5′-GGAGAAATTTTCAGAGAGAAAAA–3′ R: 5′-ATTGCTCTGCCTACAAATAAAA–3′	140–160	3	0	3	1	0.809	0.273	0.226	3	0.677	1.149
S3	F: 5′-CCCAACTCTTCGTCTATCTC–3′ R: 5′-TAGAGGTAATTGTGGGGGA–3′	100–225	5	0	5	1	0.830	0.259	0.217	5	1.083	1.702
S4	F: 5′-TTTTCCTGAATGGCATAGTT–3′ R: 5′-GCCCAATTTGTCTATCTCCT–3′	100–250	3	0	3	1	0.872	0.200	0.170	3	0.511	0.766
S5	F: 5′-CCATTGAAAACTGCACACAA–3′ R: 5′-CCATTGAAAACTGCACACAA–3′	180–600	4	0	4	1	0.803	0.299	0.249	4	0.996	1.574
S6	F: 5′-TCTTGCAATGGGGATCAG–3′ R: 5′-CGAACTATAGATAATCACTTGGAA–3′	175–550	5	0	5	1	0.936	0.118	0.111	5	0.553	0.638
S7	F: 5′-CTTCTTGAAGTTCTGGTGTTG–3′ R: 5′-ATTCTTGGAGAAAGAGTGAGG–3′	200–750	2	0	2	1	0.883	0.206	0.185	2	0.370	0.468
S8	F: 5′-ATCACCACACACTGACACAG–3′ R: 5′-CGTGTCTGAGAATCCAATATC–3′	120–190	2	0	2	1	0.777	0.345	0.285	2	0.570	0.894
S9	F: 5′-GGTGTGTTCTCTCTCTCACAC–3 R: 5′-GGGCTGCTCAATAAATGTAG–3′	150–1500	9	3	9	1	0.898	0.171	0.150	9	1.352	1.021
S10	F: 5′-ATCCTCTGCTCCTAACTTCAT–3′ R: 5′-TCTGGTACTATCCTCAAGCAA–3′	200–950	4	2	4	1	0.915	0.136	0.117	4	0.468	0.681
S11	F: 5′-ATGCCCATCTCCATATACTCT–3′ R: 5′-AATTCTTGCCTGACTCTACG–3′	200–1500	7	4	7	1	0.927	0.125	0.111	7	0.776	1.021
S12	F: 5′-GGATTCTCTAGACATGGCTTT–3′ R: 5′-AACGCAGAATTCTCTCCTACT–3′	180–200	2	0	2	1	0.564	0.486	0.368	2	0.736	1.745
S13	F: 5′-ATTGCTGTGCAATCCTTATC–3′ R: 5′-ATCTCTTTCTACCACCACGTT–3′	175–600	3	2	3	1	0.943	0.102	0.093	3	0.279	0.340
S14	F: 5′-GCAGAAGGCAATAAAGTCAT–3′ R: 5′-GCGTCAGAAGAAAAATACTGG–3′	220	1	0	1	1	0.894	0.190	0.172	1	0.172	0.213
S15	F: 5′-GGAAGGCGAGTTGATAGATAA–3′ R: 5′-CATGGGATGTTCAAAGAACT–3′	200	1	0	1	1	0.894	0.190	0.172	1	0.172	0.213
S16	F: 5′-CTTGCTTCCTCTTTTCTCTCT–3′ R: 5′-ACACTGTACTCAGCGGATTT–3′	120–900	6	0	6	1	0.830	0.272	0.230	6	1.380	2.043
S17	F: 5′-AATACCCTTCAGTATTCAGGTG–3′ R: 5′-CAACAACACAAACACTGCTAC–3′	190	1	0	1	1	0.830	0.282	0.243	1	0.243	0.340
S18	F: 5′-GGGATGTTGATAGAGATGTTG–3′ R: 5′-TCTTTCACTCTCACACACACA–3′	200	1	0	1	1	0.766	0.359	0.294	1	0.294	0.468
Total		100–1500	64	13	64	–	–	–	–	–	–	–
Mean		–	3.556	0.722	3.556	1.000	0.850	0.230	0.194	3.556	0.621	0.884
Minimum		190	1.000	0.000	1.000	1.000	0.564	0.102	0.093	1.000	0.172	0.213
Maximum		150–1500	9.000	4.000	9.000	1.000	0.943	0.486	0.368	9.000	1.380	2.043

*Na* number of alleles, *NUA* number of unique alleles, *NPA* number of polymorphic alleles, *FP* fraction of polymorphism, *FA* frequency of alleles, *GD* gene diversity, *PIC* polymorphic information content, *EMR* effective multiplex ratio, *MI* marker index, *RP* resolving power

### Gel electrophoresis

Amplified PCR samples were subjected to electrophoresis on 1.5% (for RAPD) or 3% (for SSR), agarose gel prepared in 1× TAE buffer. GeNei™ Low Range DNA Ruler plus (Cat No. 118668, 100–3000 bp) was used for RAPD, and 100 bp marker from either Promega (Cat No. G2101) or Merck Bioscience (Cat No. 61265267050173) was used for SSR, as molecular weight marker. Electrophoresis was run using 80–100 V. The gel was stained with ethidium bromide and viewed under a Gel Doc (BIO RAD Universal Hood II 2 ChemiDoc XRS, USA). The images were photographed and stored in computers. Reproducibility of the amplification products was ascertained by running at least two independent PCRs for each primer (RAPD) or primer pairs (SSR). Only consistent bands were taken for analysis.

### Statistical analysis

Each amplification product was considered as a dominant locus for RAPD and codominant locus for SSR markers. They were manually scored across all samples and recorded in the form of a binary in an excel sheet with ‘1’ for the presence of a band and ‘0’ for the absence of it. Percentage of polymorphism for each primer was derived as the proportion of polymorphic bands over the total number of different molecular weight bands observed. Marker attributes, namely, allele frequency (FA), gene diversity (GD), polymorphic information content (PIC), effective multiplex ratio (EMR), marker index (MI), and resolving power (RP), were estimated using the 3.25 version of the Power Marker statistics software (Liu and Muse 2005). Allele frequency was calculated as $\frac{{n_{u} }}{N}$, where *n*_*u*_ is the number of alleles and *N* is total number of individuals sampled. Gene diversity at the *l*th locus was estimated as $(1 - \sum_{i = 1}^{n} p_{{i^{2} }} )/(1 - \tfrac{1 + f}{N})$, where *f* is breeding incoefficient, and *pi* is the frequency of the *i*th allele. EMR was obtained by multiplying the proportion of polymorphic markers (β) and the total number of polymorphic fragments (*n*) obtained (Powell et al. 1996). The PIC refers to the value assigned to a marker in detecting an allelic variability. It is estimated as $\sum_{i = 1}^{n} p_{{i^{2} }} - 2[\sum_{i = 1}^{n - 1} \sum_{j = i + 1}^{n} p_{{i^{2} }} p_{{j^{2} }} ]$, where *pi* is the frequency of the *i*th allele (Botstein et al. 1980). The MI, signifying the overall utility of the marker system for the diversity study, was obtained as the product of PIC and EMR (Anderson et al. 1993; Varshney et al. 2007). MI together with PIC value was used to assess the informative property of the markers. Band informativeness (Ib) is given by 1 − (2 × |0.5 − *p*|), where *p* is the proportion of the total genotypes containing a particular band. It is useful in calculating the resolving power (RP), which in turn enabled us to know that the ability of a primer to distinguish various genotypes is presented as *∑*Ib (Prevost and Wilkinson 1999).

The binary data thus recorded were analyzed to obtain Jaccardʼs similarity coefficient (S) and Jaccardʼs distance (D). Subsequently, ‘S’ was used for construction of the dendrogram following UPGMA (Unweighted Pair Group Method with Arithmetic Mean) method. Principal coordinate analysis (PCoA) was done using the software PAST, version 2.15 (Hammer et al. 2001). The results were converted to a biplot to reveal the distribution pattern of the varieties in the two-dimensional space. To estimate the extent of the genetic diversity among the varieties belonging to different classes based on seed colour, the RAPD and SSR matrix data were realigned on the basis of seed coat colour and analyzed (Table 1). A dendrogram was constructed using the statistical package POPGENE 1.31, as described in Yeh et al. (1997). The binary data of RAPD and SSR were pooled to infer their combinatorial potential in revealing the genetic diversity. Pearson correlation of the variables was computed using SPSS statistical package version 16.0.2.

## Results

### DNA profile and marker attributes

Summary of binary data and estimates of the marker attributes for different RAPD primers is presented in Table 2. A representative picture of electrophoresis gel showing RAPD profile of some sesame accessions used in the study is presented in Fig. S1A. The RAPD analysis revealed a total of 256 bands of which 191 were polymorphic. The molecular weights of the amplicons ranged from 100 to 3000 bp in molecular weight. On average, 11.6 bands per primer were seen. Cent percent of polymorphic bands were obtained with primers RPI-B11 and RPI-B13, while least of the polymorphism (38.5%) was observed for RPI-B24 (Table 2). Seven of the RAPD primers amplified unique DNA fragments (Table S1). RPI-B18, RPI-B21, RPI-B22, and RPI-B23, respectively, have characterized the varieties AMRIT, YLM17, KALLIKA, and FFAT-08-22, respectively, by amplifying a unique band with each of the primers. Varieties RT46 showed amplification of three unique bands with RPI-B18 and RPI-B19, and UMA showed two unique bands with RPI-B5 and RPI-B13 (Table S1). Estimates of the marker attributes from RAPD showed a substantial difference in the potential of primers to reveal polymorphism (Table 2). Estimates of marker attributes showed that RPI-B11 primer was characterized by the highest values for GD, PIC, MI, and RP and the lowest value for FA was the most potential of the random primers in revealing polymorphism among the accessions studied. RPI-B8 was characterized by the lowest values for GD, PIC, MI, and RP but with highest value for FA. The GD that indicates the proportion of heterozygosity (*He*) was found negative correlated with FA (*r* = −0.987, *p* = 0.01). PIC, however, showed a positive correlation of 0.998 (*p* = 0.01) with GD. MI, which was maximum for the primer RPI-B11 and minimum for RPI-B8 and showed a positive correlation with PIC (0.874; *p* = 0.01).

DNA profile of the accessions using some SSR primers is shown in Fig. S1B. A total number of 64 amplicons were obtained with SSR with all of them being polymorphic (Table 3). The maximum number of nine alleles was obtained with primer S9 with molecular weights in the range of 150–1500 bp. Mean number of bands/primer obtained was 3.55. Some of the SSR primers revealed amplification of bands specific to a variety (Table S1). JLT7 and JLT26 varieties showed amplification of a unique band (allele) each with primer S1 and for RAJESWARI with the primer S10. While S13, S9, and S10 amplified two unique bands each, respectively, for CHANDANA, PRACHI, and VRI1. S11 amplified four unique bands in VINAYAK. The estimates of marker attributes for SSR primers varied widely. GD and PIC were the highest for primer S12 and least for S13 (Table 3). As observed earlier in RAPD, data here again GD showed a negative correlation of −0.971 (*p* = 0.01) with FA. PIC showed a positive correlation with GD (0.996, *p* = 0.01). The values for MI and RP were maximal for primer S16 and minimal for S14 and S15. The RP and MI one again showed a positive correlation of 0.856 with MI (*p* = 0.01).

### Cluster analysis

Matrix analysis of RAPD data showed a Jaccardʼs similarity coefficient in the range of 0.510–0.885. This reflected the presence of a high genetic variability among the accessions under study. Similarity was the highest between the accessions T13 and TKG55 and least between N32 and GT10. Clustering and PCoA confirmed this observation. UPGMA grouping reveals that the sesame germplasm under study can be divided into two distinct clusters (Fig. S2A). Cluster A was smaller and comprised of 11 accessions. Cluster B was larger which diverged further into two subclusters, namely, B1 and B2. Cluster B1 further subdivided into B1a comprising of 24 accessions and B1b comprising of 11 varieties. Cluster B2 was represented by solitary TC289.

Jaccards similarity coefficient obtained from SSR data ranged from 0.167 to 0.867, indicating a high genetic variability among the accessions. In contrast to RAPD, SSR reveals that RT127 and JT7 were the closest and PRACHI and YLM17 were highly dissimilar. The UPGMA of SSR data grouped the accession into two clusters (Fig. S2B). There was complete realignment of the varieties observed. Cluster A was represented by the solitary PRAGHTI. Remaining 46 accessions together formed cluster B, which further got split into B1 and B2 subclusters. Cluster B1 was the largest with 40 accessions, and cluster B2 consisted of six cultivars. The cophenetic correlation was 0.8 for both RAPD and SSR clusters which was considered as a good fit for the data matrix and the resultant clusters (Romesburg 1990).

**Fig. 1:**
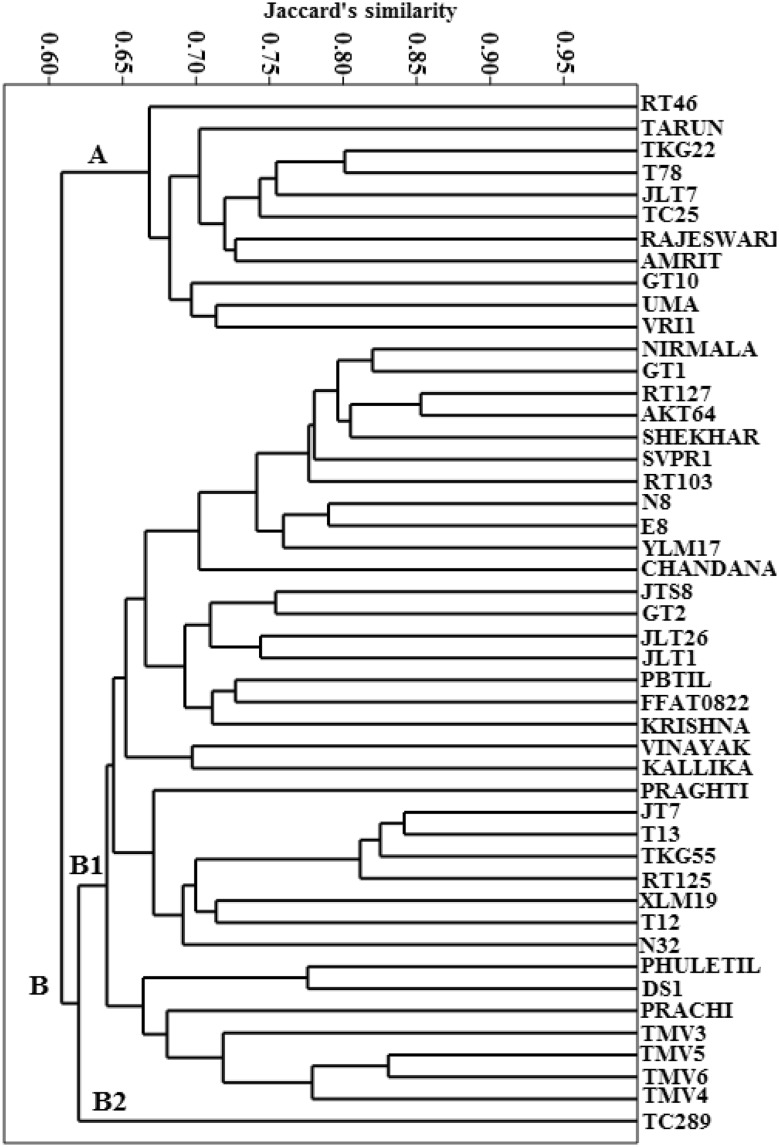
UPGMA based dendrogram of the germplasm of *Sesamum indicum* L. based on pooled data of RAPD and SSR markers

Dendrogram obtained after pooling of the RAPD and SSR binary data is presented in Fig. 1. The polymorphism now observed was 79.7% which was little higher as compared to polymorphism obtained with RAPD. Jaccardʼs similarity coefficient ranged from 0.505 to 0.853. The most diverse varieties, however, were N32 and JLT7, and the closest were AKT64 and RT127. The gross structure of dendrogram and grouping of the accessions into clusters showed a similarity with the distribution obtained with that of RAPD data. The accessions were observed to realign into two major clusters. Cluster A consisted of 11 genotypes. Cluster B subdivided into B1 comprising of 36 genotypes and TC289 remaining as solitary under cluster B2 as in RAPD.

### Principal coordinate analysis

The PCoA of RAPD data grouped the accession in a two-dimensional space with a clustering pattern synonymous with the dendrogram obtained with RAPD (Fig. S3A). Analysis revealed that the first two principal coordinates accounted for about 30% of the total variance. PCoA divided the cultivars into three groups. Group A included 24 varieties (T12, RT125, XLM19, T13, TKG55, N32, JT7, PRAGHTI, TMV3, TMV4, TMV5, TMV6, PRACHI, VINAYAK, PBTIL, JTS8, FFAT0822, KRISHNA, GT2, JLT26, JLT1, PHULETIL, TC289, and DS1). Group B consisted of 11 varieties (RAJESWARI, JLT7, TC25, RT46, TKG22, UMA, T78, TARUN, GT10, AMRIT, and VRI1). Group C consisted of 12 varieties (YLM17, E8, N8, RT103, AKT64, RT127, SHEKHAR, SVPR1, NIRMALA, GT1, CHANDANA, and KALLIKA).

For SSR data, the PCoA showed that the first two principal coordinates accounted for about 25% of the total variance. However, there observed a complete realignment of the accessions leading to clustering of accessions into three groups (Fig. S3B). Group A consisted of 34 varieties (NIRMALA, JLT1, RT103, PRAGHTI, SVPR1, RT46, TMV4, TMV6, TMV3, SHEKHAR, T13, PHULETIL, GT2, AMRIT, KALLIKA, TC289, TKG55, AKT64, GT1, GT10, CHANDANA, TARUN, T78, UMA, PBTIL, VINAYAK, E8, N8, N32, TC25, RT125, XLM19, DS1, and JLT7). Group B consisted of seven varieties (RAJESWARI, T12, JLT26, PRACHI, VRI1, KRISHNA, and TMV5). In addition, Group C included six varieties (FFAT0822, RT127, JTS8, TKG22, YLM17, and JT7).

**Fig. 2:**
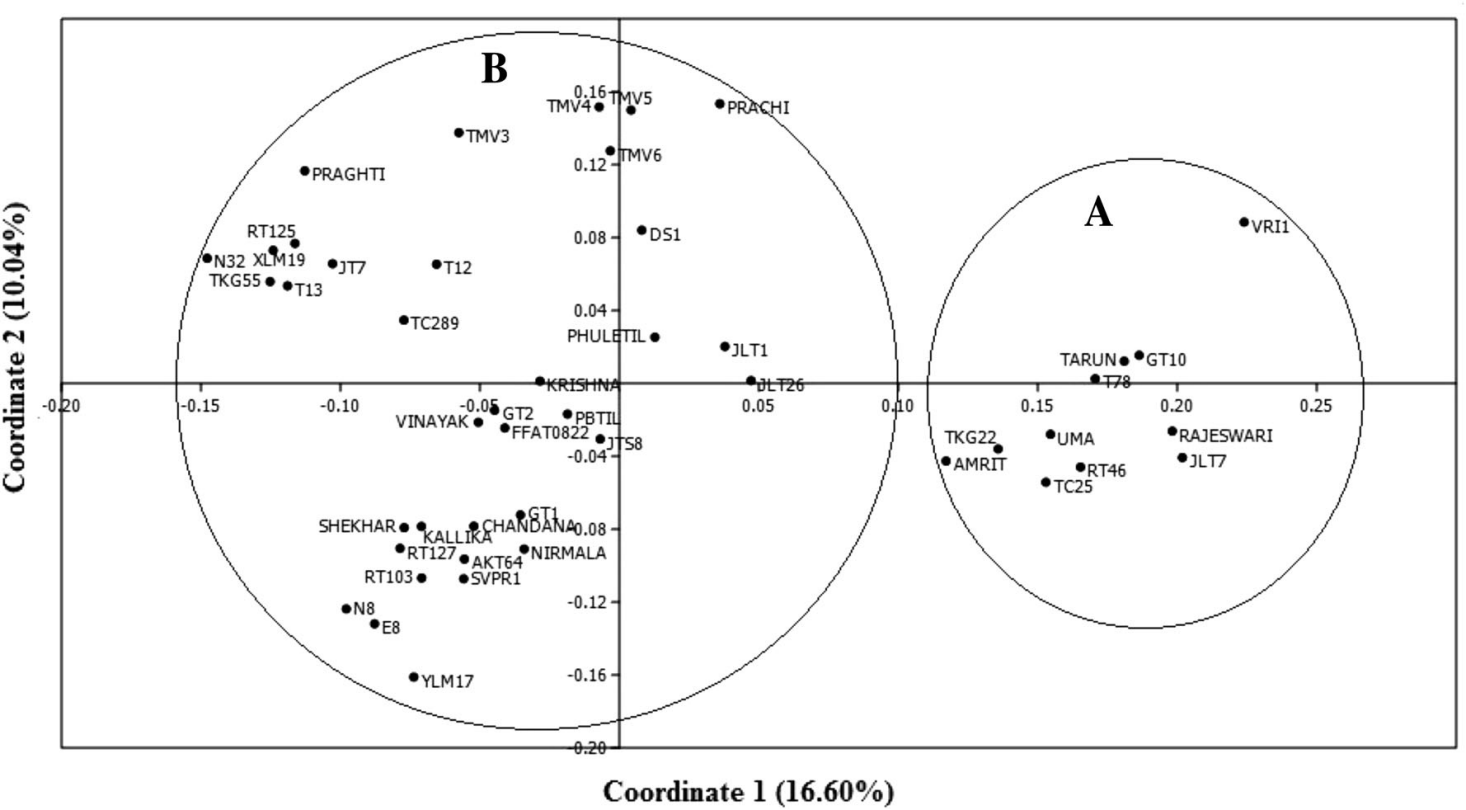
Principal coordinate analysis of *S.indicum* accessions in two-dimensional space based on pooled data of RAPD and SSR markers

PCoA of pooled data (RAPD and SSR) gave a grouping pattern that resembled the one obtained with RAPD data as seen earlier with dendrogram plots. There were two distinct major groups observed. Group A had 11 varieties, and Group B consisted of 36 accessions, including TC289 that remained solitary in dendrogram (Fig. 2).

### Genetic diversity among and within the population on the basis of seed colour

There were 29 white, 13 brown, and 5 black seeded varieties recognized in the population (Table 1). Analysis of the molecular data for the seed coat colour revealed that of a set of markers could be recognized that correlate with seed colour. The trend was applicable for both RAPD and SSR markers. Black varieties were characterized by complete absence of specific amplicons in 35 out of 45 instances for RAPD and in 25 out of 35 instances in SSR profiles (Tables S2, S3). The proportion of genetic diversity (Gst) observed between seed colour populations was 9% in the case of RAPD and 7% in the case of SSR. POPGENE analysis and tree construction revealed that white and brown seeded populations were close to each other as compared to the black seeded population (Fig. S4).

## Discussion

Sesame is an unexplored crop which needs a focused research for its genetic improvement in several fronts. Major sesame traits that require attention to achieve higher yields are harvest index, seed retention, uniform maturity, and resistance to abiotic and biotic stresses. Foremost requirement for this is the assessment of genetic diversity available in the crop. RAPD and SSR markers have been effectively used for the assessment of molecular diversity, and the data were integrated with the other markers for construction of genetic map in several crop plants (Nimmakayala et al. 2005). In the present study, with the use of two markers, we demonstrate the presence of a considerable genetic variability in the germplasm of sesame grown in different agroclimatic zones of India.

The extent of polymorphism and diversity reported herein is comparable to genetic diversity reported for certain varieties of sesame grown in India as well as for germplasm from other countries, such as Turkey (Ercan et al. 2004; Frary et al. 2015; Uncu et al. 2015) and Cambodia and Vietnam (Pham et al. 2009). Up to 73% polymorphism was reported for core collection of sesame from China (Zhang et al. 2010) and for Indian genotypes (Kumar and Sharma 2011). Three of the primers viz., RPI-B5, RPI-B17, and RPI-B19 that were common to some of the previous studies gave similar results (Bhat et al. 1999; Pham et al. 2009). Normally, SSR markers are codominant in nature and help in identification of heterozygotes in the population. In the present study, however, the SSR gave a higher mean value for alleles per locus indicating the presence of multiple loci for certain alleles in some of the varieties.

Variability in marker attributes resulted from the differences in primers used and their priming sites. The RAPD primer RPI-B11 and the SSR primer S16 were found to be the most informative primers to discriminate the sesame genotypes. A higher value for the marker attributes is critical for considering use of the dominant (RAPD and AFLP) and codominant markers (SSR and RFLP) for classification, fingerprinting, genetic diversity analysis, gene mapping, molecular breeding, and germplasm evaluation (Sathyanarayana et al. 2011). On comparative basis, we found that GD was almost similar for both RAPD (0.229) and SSR (0.230). Mean values of MI and RP were larger in case of for RAPD as compared to SSR. Mean PIC value, however, was found to be larger for SSR (0.194) than RAPD (0.186). The higher PIC value for SSR has been reported earlier in common bean (Zargar et al. 2016) and walnut (Ahmed et al. 2012). The PIC values ranging from 0.03 to 0.96 were reported in the previous studies also (Li-Bin et al. 2008; Cho et al. 2011; Spandana et al. 2012; Park et al. 2013).

The genetic similarity coefficient observed in our study (0.510–0.885 in case of RAPD; 0.167–0.867 in case of SSR; and 0.503–0.853 in case of pooled data of RAPD and SSR) was comparable with earlier reports (Bhat et al. 1999; Kumar and Sharma 2009; Yepuri et al. 2013). It is a general practice to use a dominant (RAPD) and a codominant (SSR) markers for diversity studies. The clustering pattern obtained with the pooled RAPD and SSR binary data reassembled the one based on RAPD data alone as has been reported recently in bamboo (Desai et al. 2015). The impact of RAPD data may be attributed due to a higher number of markers involved in analysis as compared to that of the SSR. This led us to conclude that high resolution of crop genotypes into a phylogeny can be achieved considerably with higher number of polymorphic marker. The unique bands amplified by RAPD and SSR primers are important as they could be developed into PCR-based SCAR and sequence-tagged CAPS markers, respectively, for use in fingerprinting, identification of interspecific hybrids, marker-assisted selection for crop improvement, and genetic resource management (Fernadez et al. 2002; Xu 2003; Bandyopadhyay 2011).

Seed coat colour is an important trait that is associated with properties, such as antioxidant activity and disease resistance exhibited by sesame seed. The seed coat trait also finds application in estimating sesame evolution (Zhang et al. 2013a). In this study, we reported that white and brown population was phylogenetically close as compared to black one. We also found that at molecular level, the black varieties had the absence of specific DNA bands rather than its presence in white and brown varieties. Occurrence of three different seed colours indicates that this trait may be controlled by more than one gene as has been recently reported in sesame by Zhang et al. (2013a) and in other crops (Padmaja et al. 2005). Genetic diversity among the three populations was estimated to be lesser as compared to within population variability and such variability could be of use in genetic improvement by selection within groups and without having to compromise on seed coat colour.

We conclude that the polymorphism and fine marker attribute reported herein can be reliably used for the selection and evaluation of parents in sesame breeding programs, maintenance of germplasm, DNA fingerprinting, and analyzing the evolutionary path of sesame cultivars. Further the data would be useful in identifying parents for heterosis breeding, conservation, and genetic improvement for yield and other desirable traits in sesame. The sesame genome sequence recently studied by Zhang et al. (2013b) has provided and opened up new challenges for the present scientists to improve the crop efficiently at each and every level.

## Electronic supplementary material

Below is the link to the electronic supplementary material.

Supplementary material 1 (DOCX 698 kb)
