# Supplementary material for: Comparative assessment of genetic diversity in Sesamum indicum L. using RAPD and SSR markers
Source: 3 Biotech. 2017 Apr 8;7(1):10. doi: 10.1007/s13205-016-0578-4 (PMC5385169; doi:10.1007/s13205-016-0578-4)
Supplement: Supplementary file 1 — Supplementary material 1 (DOCX 698 kb) [file 13205_2016_578_MOESM1_ESM.docx]

**Supplementary material**

**
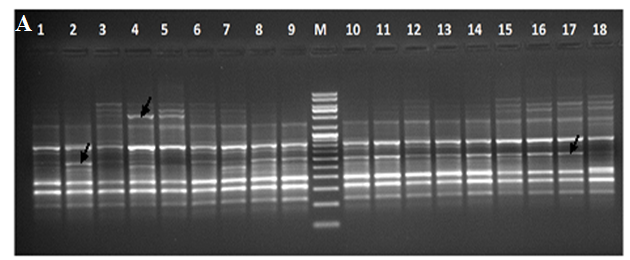
**

**
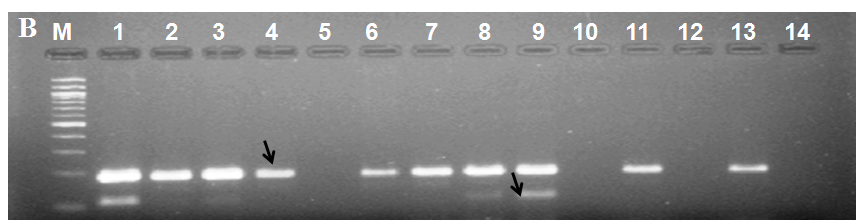
**

**Figure S1** DNA profile of some of the *Sesamum indicum* accessions with RAPD and SSR primers (arrows point the polymorphic bands). **A** is with a RAPD primer RPI-B25. Lanes 1–18 correspond to the sesame accessions VRI1, UMA, GT10, TARUN, PHULETIL, JLT7, AMRIT, RT46, RAJESWARI, T78, TC25, TKG22, NIRMALA, GT1, SHEKHAR, AKT64, RT127 and SVPRI. Lane M is the standard low range 100–3000 bp DNA ruler. **B** is with a SSR primer S8. Lanes 1–14 correspond to RT125, NIRMALA, RT103, RAJESWARI, SVPR1, VINAYAK, E8, T12, PRACHI, T78, N32, TMV3, TMV5 and TARU N. Lane M is 100 bp marker


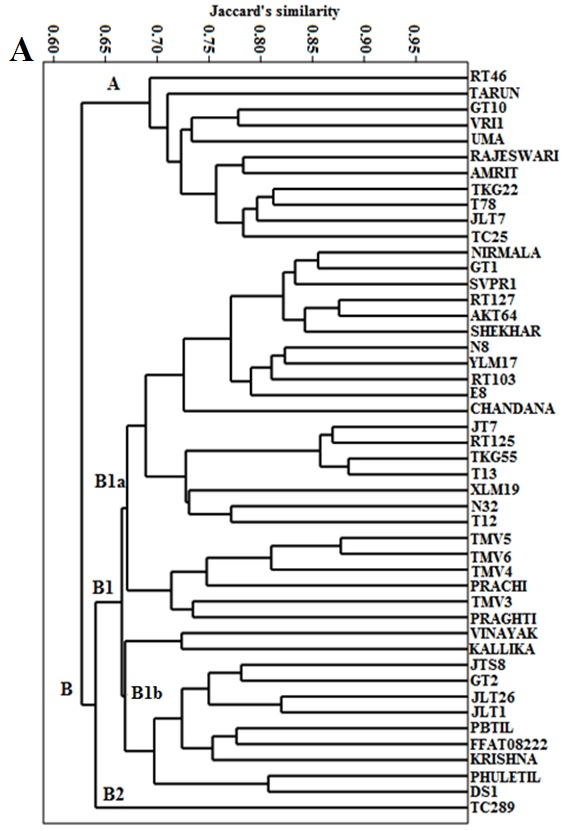


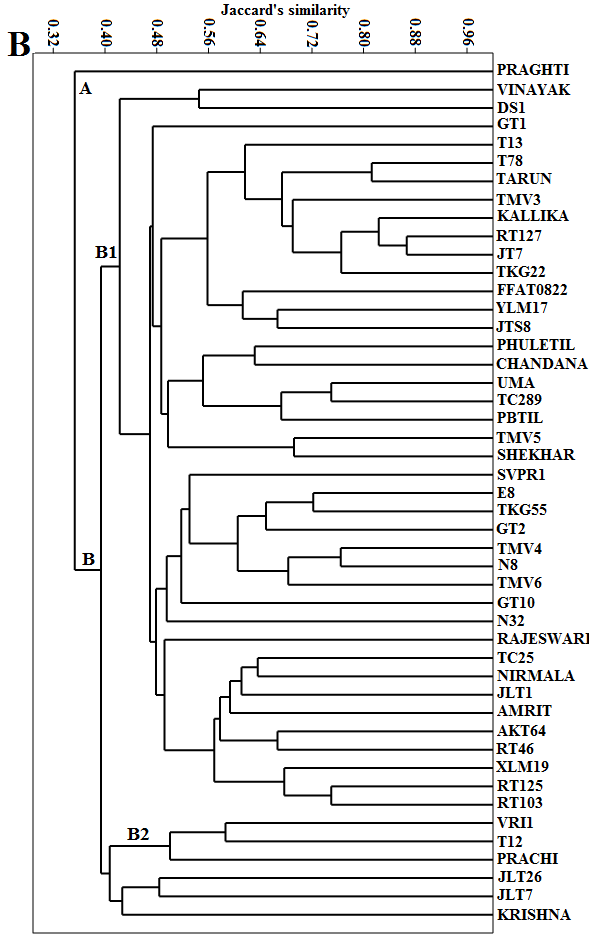


**Figure S2** UPGMA based dendrogram of the germplasm of *Sesamum indicum* L. **A** is based on RAPD markers, **B** is based on SSR markers


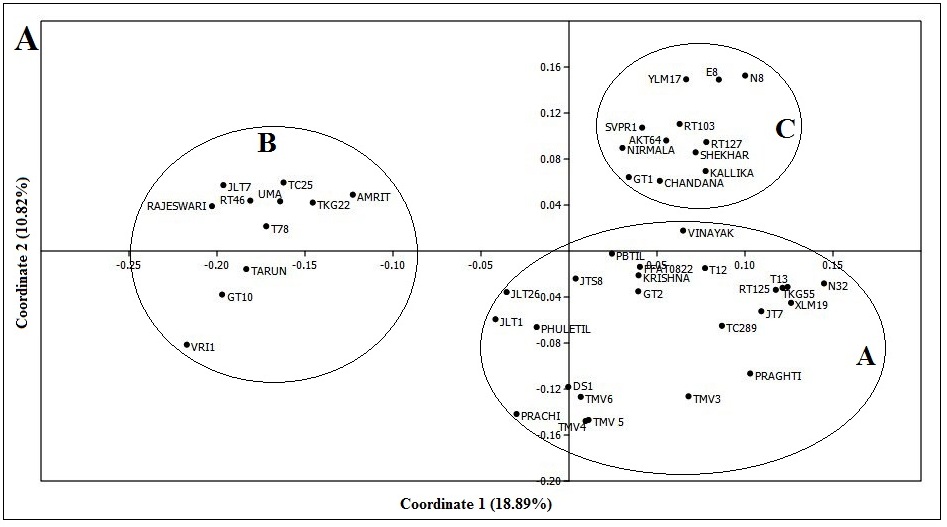


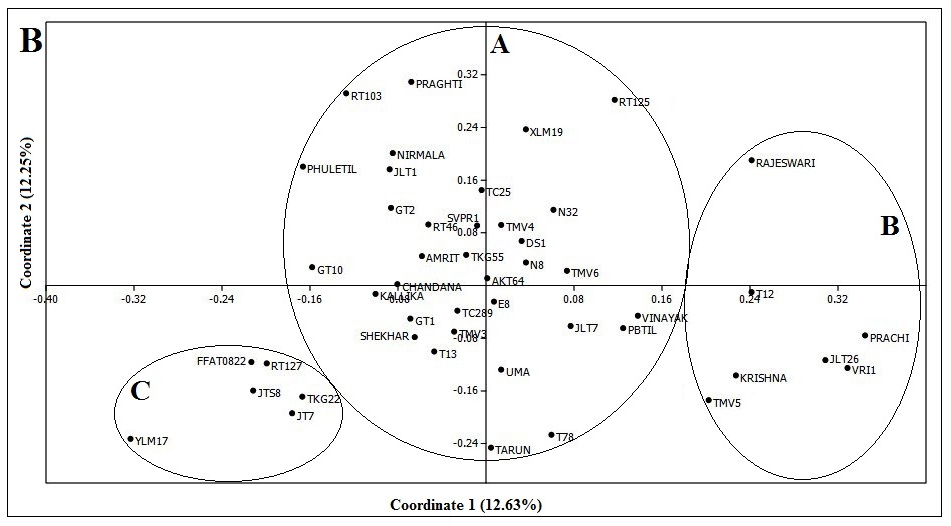


**Figure S3** Principal coordinate analysis of *S.indicum* accessions in two dimensional space. **A** based on RAPD markers, **B** based on SSR markers


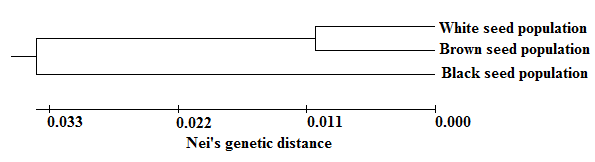


**Figure S4** Phylogenetic tree illustrating genetic relationship among three seed coat colour populations.

**Table S1** Details of primers revealing unique band pattern in *Sesamum indicum* L.

| Primer | NUA | Amplicon size (bp) | Variety |
| --- | --- | --- | --- |
| RPI-B5 | 1 | 2000 | UMA |
| RPI-B13 | 1 | 1100 | UMA |
| RPI-B18 | 2 | 1400 | RT46 |
|  |  | 1500 | AMRIT |
| RPI-B19 | 2 | 600 | RT46 |
|  |  | 1250 | RT46 |
| RPI-B21 | 1 | 900 | KALLIKA |
| RPI-B22 | 1 | 300 | YLM17 |
| RPI-B23 | 1 | 100 | FFAT0822 |
| S1 | 2 | 100 | JLT7 |
|  |  | 400 | JLT26 |
| S9 | 3 | 220 | PRACHI |
|  |  | 500 | RAJESWARI |
|  |  | 850 | PRACHI |
| S10 | 2 | 750 | VRI1 |
|  |  | 950 | VRI1 |
| S11 | 4 | 300 | VINAYAK |
|  |  | 490 | VINAYAK |
|  |  | 510 | VINAYAK |
|  |  | 1500 | VINAYAK |
| S13 | 2 | 175 | CHANDANA |
|  |  | 600 | CHANDANA |

RPI-B: RAPD primers, S: SSR primers

**Table S2** Data on specific RAPD primer-marker combination associated with seed coat colour in *S. indicum*

| Primer | Marker size (kbp) | Percentage of varieties amplifying the marker | | |
| --- | --- | --- | --- | --- |
|  |  | White | Brown | Black |
| RPI-B3 | 0.90 | 20.69 | 15.38 | 0.00 |
|  | 2.50 | 13.79 | 23.08 | 0.00 |
| RPI-B4 | 0.80 | 17.24 | 23.08 | 0.00 |
|  | 1.75 | 10.34 | 7.69 | 0.00 |
| RPI-B5 | 0.75 | 27.59 | 15.38 | 0.00 |
|  | 1.00 | 0.00 | 23.08 | 40.00 |
|  | 1.30 | 3.45 | 15.38 | 0.00 |
|  | 2.00 | 0.00 | 7.69 | 0.00 |
| RPI-B7 | 0.20 | 10.34 | 15.38 | 0.00 |
| RPI-B9 | 0.30 | 10.34 | 15.38 | 0.00 |
|  | 0.39 | 17.24 | 7.69 | 0.00 |
|  | 1.00 | 0.00 | 7.69 | 20.00 |
| RPI-B10 | 0.28 | 13.79 | 7.69 | 0.00 |
|  | 1.50 | 3.45 | 0.00 | 20.00 |
|  | 1.75 | 17.24 | 0.00 | 20.00 |
| RPI-B12 | 0.65 | 13.79 | 0.00 | 40.00 |
| RPI-B13 | 0.38 | 17.24 | 0.00 | 0.00 |
|  | 0.65 | 10.34 | 23.08 | 0.00 |
|  | 1.10 | 0.00 | 7.69 | 0.00 |
|  | 3.00 | 6.90 | 7.69 | 0.00 |
| RPI-B14 | 0.75 | 17.24 | 15.38 | 0.00 |
|  | 1.30 | 13.79 | 15.38 | 0.00 |
| RPI-B15 | 0.35 | 3.45 | 7.69 | 0.00 |
|  | 1.10 | 17.24 | 0.00 | 0.00 |
| RPI-B17 | 0.52 | 24.14 | 0.00 | 0.00 |
|  | 0.61 | 6.90 | 0.00 | 0.00 |
|  | 0.70 | 31.03 | 15.38 | 0.00 |
|  | 1.20 | 31.03 | 23.08 | 0.00 |
| RPI-B18 | 1.40 | 3.45 | 0.00 | 0.00 |
|  | 1.50 | 0.00 | 7.69 | 0.00 |
| RPI-B19 | 0.61 | 3.45 | 0.00 | 0.00 |
|  | 0.81 | 13.79 | 0.00 | 0.00 |
|  | 1.25 | 3.45 | 0.00 | 0.00 |
| RPI-B20 | 0.58 | 24.14 | 0.00 | 0.00 |
|  | 0.90 | 0.00 | 15.38 | 40.00 |
|  | 1.50 | 3.45 | 7.69 | 0.00 |
| RPI-B21 | 0.30 | 0.00 | 7.69 | 20.00 |
|  | 0.50 | 6.90 | 23.08 | 0.00 |
|  | 0.89 | 0.00 | 7.69 | 0.00 |
| RPI-B22 | 0.30 | 0.00 | 7.69 | 0.00 |
|  | 1.50 | 31.03 | 30.77 | 0.00 |
| RPI-B23 | 0.10 | 3.45 | 0.00 | 0.00 |

RPI-B1‒23 RAPD primer set

**Table S3** Data on specific SSR primer‒marker combination associated with seed coat colour in *S. indicum*

| Primer | Marker size (kbp) | Percentage of varieties amplifying the marker | | |
| --- | --- | --- | --- | --- |
|  |  | White | Brown | Black |
| S1 | 0.10 | 3.45 | 0.00 | 0.00 |
|  | 0.25 | 10.34 | 0.00 | 20.00 |
|  | 0.40 | 3.45 | 0.00 | 0.00 |
|  | 0.60 | 13.79 | 0.00 | 0.00 |
|  | 0.70 | 13.79 | 7.69 | 0.00 |
| S2 | 0.14 | 3.45 | 7.69 | 0.00 |
|  | 0.16 | 3.45 | 7.69 | 0.00 |
| S3 | 0.12 | 6.90 | 7.69 | 0.00 |
|  | 0.19 | 3.45 | 0.00 | 40.00 |
| S4 | 0.10 | 6.90 | 7.69 | 0.00 |
|  | 0.12 | 0.00 | 7.69 | 20.00 |
| S5 | 0.60 | 10.34 | 0.00 | 20.00 |
| S6 | 0.17 | 10.34 | 0.00 | 0.00 |
|  | 0.40 | 3.45 | 7.69 | 0.00 |
|  | 0.55 | 3.45 | 7.69 | 0.00 |
| S9 | 0.15 | 31.03 | 0.00 | 0.00 |
|  | 0.19 | 0.00 | 7.69 | 20.00 |
|  | 0.22 | 0.00 | 0.00 | 20.00 |
|  | 0.40 | 6.90 | 0.00 | 20.00 |
|  | 0.50 | 3.45 | 0.00 | 0.00 |
|  | 0.60 | 3.45 | 0.00 | 20.00 |
|  | 0.85 | 0.00 | 0.00 | 20.00 |
| S10 | 0.20 | 6.90 | 0.00 | 0.00 |
|  | 0.75 | 0.00 | 7.69 | 0.00 |
|  | 0.95 | 0.00 | 7.69 | 0.00 |
| S11 | 0.30 | 0.00 | 7.69 | 0.00 |
|  | 0.49 | 0.00 | 7.69 | 0.00 |
|  | 0.51 | 0.00 | 7.69 | 0.00 |
|  | 0.60 | 6.90 | 7.69 | 0.00 |
|  | 0.90 | 13.79 | 23.08 | 0.00 |
|  | 1.50 | 0.00 | 7.69 | 0.00 |
| S13 | 0.17 | 0.00 | 7.69 | 0.00 |
|  | 0.60 | 0.00 | 7.69 | 0.00 |
| S16 | 0.25 | 10.34 | 23.08 | 0.00 |
|  | 0.90 | 0.00 | 0.00 | 40.00 |

S1‒S16 SSR primer set
